# Characteristics of meat production traits in the Hungarian Simmental herd selected for the simultaneous improvement of milk and meat production

**DOI:** 10.5194/aab-66-233-2023

**Published:** 2023-09-08

**Authors:** József Péter Polgár, Ferenc Szabó, Ákos Kovács, Zoltán Kovács-Mesterházy, Szabolcs Bene

**Affiliations:** 1 Institute of Animal Sciences, Georgikon Campus, Hungarian University of Agriculture and Life Sciences, Keszthely, Hungary; 2 Department of Animal Sciences, Albert Kázmér Faculty of Mosonmagyaróvár, Széchenyi István University, Győr, Hungary; 3 Association of Hungarian Simmental Breeders, Bonyhád, Hungary

## Abstract

Phenotypic and genetic trends, population genetic parameters, and the heritability and breeding values of the fattening and slaughter traits – namely muscularity score (MUS), final fattening weight (FFW), weight gain per day of life (WGD), slaughter weight (SLW), carcass weight (CAR), dressing percentage (DRP), bone–meat production per day of life (BMP), SEUROP conformation and fat coverage score (EUR, FAT), and meat percentage (MEP) – of 1162 Hungarian Simmental bulls were evaluated with the progeny test database of the Association of Hungarian Simmental Breeders. Trends were calculated by weighted linear regression analysis, while the population genetic parameters and breeding values used the BLUP (best linear unbiased prediction) animal model and general linear model (GLM). According to the results, the lowest heritability value ( $h^{2}=0.23$ ) was found in FAT. In the case of MUS, EUR and MEP, the values were moderate ( $h^{2}=0.32$ , 0.26 and 0.32), and in the case of the other traits, high heritability values ( $h^{2}$ = 0.42–0.52) were estimated. The phenotypic trends of the fattening and slaughter traits of bull progeny born between 2001 and 2019 showed a stagnant direction. Between the sires, the differences in the breeding values for some traits (WDG, CAR and BMP) were large, and with other traits (DRP, EUR and FAT), small differences were found. According to the data of the genetic-trend calculation, the steepness values of the evaluated traits were positive, and the genetic trends showed a slightly improving direction in the estimated period.

## 1 Introduction and literature review

In the selection of the Hungarian Simmental breed, there is a double difficulty. Due to the simultaneous selection of milk and meat production, the number of traits taken into account can definitely be more diverse than that in the case of specialized breeds. As a result of this, along with the genetic antagonism between milk and meat production traits, the extent and assessment of genetic progress achieved in some traits may differ from what is usual for specialized breeds. In the case of dual-purpose breeds, such as the Hungarian Simmental, it can be considered to be a good result if, in addition to achieving considerable genetic progress in a dairy trait, we do not experience a decrease in some beef traits.

In the Hungarian Simmental breed, 12 traits (3 of these were meat production traits, namely weight gain per day of life, meat percentage and SEUROP conformation score) were marked to be part of its breeding purpose and to be improved upon. The mentioned traits were included in the selection index, which is called the dual-purpose production index (DPI). During the formation of the selection index, the breeding values estimated for the given traits are considered as a starting point; these traits are determined from the data obtained during the progeny tests. Therefore, breeding bulls suitable for artificial insemination take part in a progeny test, during which a breeding value is estimated for the production (milk, meat, fitness) and conformation characteristics.

Consequently, progeny tests organized to assess the meat-producing ability were separated from other types of fattening or slaughter experiments, such as breed comparison tests; however, the scope of the examined traits (weight gain, final fattening weight, slaughter %, etc.) can be very similar in these two test systems. In the literature, we can find many studies (Holló et al., 2008; Özlütürk et al., 2004; Geuder et al., 2012; Bureš and Bartoň, 2018, etc.) whose results were obtained under experimental conditions, but the number of results based on the progeny performance test is extremely small.

There were a number of sources in the literature regarding the heritability of fattening and slaughter characteristics of different breeds of cattle. According to Crews et al. (2003), the heritability ( $h^{2}$ ) values of the slaughter weight and the carcass weight were 0.47 and 0.53, respectively, in the Simmental breed. Su et al. (2017) reported a value of 0.34 for the heritability of carcass weight. Hickey et al. (2007) found that the heritability of the SEUROP fat coverage score was low (0.17–0.26) when evaluating a multi-breed database. Rumph et al. (2007) showed that the heritability of carcass traits of the Simmental-sired fattening bulls ranged from 0.12 to 0.34. According to Engellandt et al. (1999), the heritability of the dressing percentage of Gelbvieh bulls was 0.50. Evaluating a database of different breeds, Coyne et al. (2019) found a similar heritability value of 0.48 for the dressing percentage. The heritability of the muscularity score determined by Cesarani et al. (2020) was small (0.23) in the Italian Simmental herd.

The meat-producing capacity, i.e., the growth rate, weight gain during fattening and slaughter results, apart from genetic background, can be greatly influenced by different environmental effects (Vorisková et al., 2002; Pogorzelska-Przybylek et al., 2018). Numerous literature sources on the study of environmental factors that influence fattening and slaughter values have been published (Gregory et al., 1994; Steen, 1995; Laborde et al., 2001; Bjelka et al., 2002). The results of these sources have been presented in detail in our previous work (Polgár et al., 2005) so they are not detailed here.

Very little novel data on the phenotypic and genetic trends in fattening and slaughter traits have been found in the literature (Emmerling et al., 2019). For the Simmental breed and the Simmental-type Bavarian Spotted breed, Elzo et al. (1987) and Kögel et al. (1991), respectively, reported data, but these can be considered to be quite old. Potočnik et al. (2007) identified increasing genetic trends in a number of traits related to milk production and in the conformation and slaughter traits in Slovenian Simmental herds. Kapš et al. (2000) did not show a trend-like change when evaluating the change in the weight gain per day of life of fattening bulls in the progeny test. Similar results have been reported by Röhrmoser and Pichler (2002).

Since relatively little information is available in the literature on the subject in question, the aim of our present study was

- to determine the effect of different factors on the fattening and slaughter traits
- to determine the heritability values of the mentioned traits
- to determine the breeding values of sires based on the mentioned traits
- to determine the direction of the phenotypic and genetic trend in the mentioned traits over the past 20 years
- to determine the changes in the meat production of the dual-purpose Hungarian Simmental breed as a result of the simultaneous selection aimed at improving both milk and meat production.

## 2 Materials and methods

This research is based on the results obtained in our two previous studies (Polgár et al., 2016; Bene et al., 2016). Therefore, there were many similarities in the structure of the database, as well as in the methodological parts, which we will not repeat in the present paper.

### 2.1 The progeny test and the database

In this study, the results of purebred male progeny of 111 dual-purpose Hungarian Simmental sires (the data of meat-type sires and their offspring were not included in the evaluation) were processed. A total of 1162 bull progeny of the 111 sires from 1023 randomly selected dams were included in the study. The bull progeny were fattened and then slaughtered according to the progeny test protocol of the Association of Hungarian Simmental Breeders.

The number of offspring per sire ranged from 5 to 31, with an average of 10.5 offspring per sire. The bull progeny were born between 8 January 2001 and 8 December 2019, and the slaughter took place between 13 May 2002 and 3 May 2021. This rather long period gave us the opportunity to evaluate the phenotypic and genetic trends.

The youngest bull progeny in the test was slaughtered at 12 months of age, and the oldest one was slaughtered at 27 months of age. The average slaughter age was 18.1 months. Of the 111 dual-purpose sires in the study, the oldest were born in 1995, and the youngest were born in 2016; thus, we were able to calculate genetic trends for the period 1995–2016.

A total of 10 fattening farms took part in the progeny-testing procedure. The procedure for organizing and conducting the progeny performance test and the conditions for the keeping and feeding of fattening bulls were described in Füller et al. (2009) and so are not repeated here.

Data collection was regularly monitored by the staff of the Association of Hungarian Simmental Breeders. The measurements (live-weight measurement before slaughter, measurement of carcasses after slaughter, etc.) and data collection were carried out using the same methods and at the same times in terms of slaughter and cutting technology at the different slaughterhouses.

The Kolmogorov–Smirnov test was used to check the normal distribution of the database, and the Levene test was used to check the homogeneity of the variances.

### 2.2 The examined traits

The evaluated traits were as follows: muscularity score (MUS), final fattening weight (FFW), weight gain per day of life (WGD), slaughter weight (SLW), carcass weight (CAR), dressing percentage (DRP), bone–meat production per day of life (BMP), SEUROP conformation and fat coverage score (EUR, FAT), and meat percentage (MEP). The method of calculation, the acronyms and the units of measurement of the examined traits are summarized in Table 1.

**Table 1:** The evaluated traits.

Traits	Sign	Unit	Calculation and measuring method (description)
Muscularity score	MUS	point	Individually rated under the conformation scoring
Final fattening weight	FFW	kg	Individual live weight measured at the end of fattening in fattening farm
Weight gain per day of life	WGD	g d ${}^{-1}$	FFW/age of bull at slaughter (SLA) × 1000
Slaughter weight	SLW	kg	Individual live weight measured in the slaughterhouse before slaughter
Carcass weight	CAR	kg	Individual carcass weight after the splitting
Dressing percentage	DRP	%	CAR / SLW × 100
Bone–meat production per day of life	BMP	g d ${}^{-1}$	CAR / SLA × 1000
SEUROP conformation score	EUR	point	Individual SEUROP conformation score of carcass in five point categories, from P at 1 point to E at 5 points
SEUROP fat coverage score	FAT	point	Individual SEUROP fatness score of carcass in five point categories, from 1 = 1 point to 5 = 5 points
Meat percentage	MEP	%	Meat / CAR × 100

### 2.3 Examining the effect of different factors

Data were evaluated by a multivariate analysis of variance (general linear model – GLM). In constructing the model, the sire of the fattening bulls was taken into account randomly; the location of the fattening farm (the fattening farm from which the offspring went to the slaughterhouse) and the year of birth of the bull progeny were considered as fixed effects (Gregory et al., 1995; Lee et al., 1997). The slaughter age (SLA) of the bull progeny was included in the model as a covariant. In the course of the study, the 10 traits mentioned above were handled separately from each other, and model calculations (runs) were performed separately.

The general form of the estimation models used (using the CAR as an example) is described as follows:

$${\hat{y}}_{hijk}=\mu +S_{h}+F_{i}+Y_{j}+b(x_{hijk}-X)+e_{hijk},$$

where ${\hat{y}}_{hijk}$ is the CAR of bull progeny of age k , born in year j and fattened from sire h in place i ; μ is the mean of all observations; $S_{h}$ is the random effect of sire; $F_{i}$ is the fixed effect of the place of fattening; $Y_{j}$ is the fixed effect of year of birth; b is the regression coefficient (slaughter age); and $e_{hijk}$ is the random error.

For all traits, the significance of the above-mentioned effects was also studied, but in the present paper, only the results for the sire and the birth year of the bull progeny are presented.

Phenotypic correlation coefficients were determined between the studied traits.

The data were prepared using Microsoft Excel 2003 and Word 2003. The evaluation of the database was performed with the statistical software package SPSS 27.0 (IBM Corporation, 2020).

### 2.4 Phenotypic trends

When calculating phenotypic trends, we started with the average results of bull progeny per year of birth. Linears were fitted to the annual mean of the studied traits using one-way linear regression analysis. The evaluated traits were considered to be dependent variables, and the year of birth was considered to be an independent variable. The general form of the one-way linear regression equation used was as follows:

Y=a+bX,

where Y is the phenotypic mean value of the measured trait; a is the intercept; b is the slope, the magnitude of change and the direction of the trait; and X is the year of birth of the fattening bull participating in the progeny performance test.

### 2.5 Estimation of population genetic parameters

Calculation of the population genetic parameters took place in accordance with the work of Szőke and Komlósi (2000) and Lengyel et al. (2004), with two models being used, namely one GLM model and one BLUP (best linear unbiased prediction) animal model.

- The GLM model (ANOVA Type III) was completely identical to the model presented in Sect. 2.3; thus, we will not repeat it here.
- Using the BLUP models, two matrices were created. One of these was the database matrix, and the other was the pedigree matrix. The pedigree matrix of relatives included pedigree data for full sibs, half sibs, sires and dams.

The examined fix (environmental) factors for all models were the fattening farm and the year of birth of the bull progeny. The covariant was SLA in the models.

Over the course of the study, four population genetic values were determined for each trait with both models:

- the genetic variance ( $\sigma_{d}^{2}$ ) among the progeny groups
- the residual (environmental) variance ( $\sigma_{e}^{2}$ ) within the progeny groups
- the phenotypic variance ( $\sigma_{p}^{2}=\sigma_{d}^{2}+\sigma_{e}^{2}$ )
- the heritability value ( $h^{2}=\sigma_{d}^{2}/\sigma_{p}^{2}$ ).

The method of calculation of the population genetic parameters was described in detail in our previous paper (Bene et al., 2020); thus, it is not detailed here.

For the GLM model, SPSS 27.0 (IBM Corporation, 2020) software was used, and for the BLUP animal model, DFREML (Meyer, 1998) and MTDFREML (Boldman et al., 1993) software were used.

### 2.6 Estimation of breeding values

The breeding value (BV) of the sires in the study was also estimated with two models based on the evaluated traits.

In case of the GLM method, the BV was determined as the difference between the average performance of the sire's progeny group and the average performance of the entire population for all 10 traits:

$$\mathrm{BV}=(x_{p}-X_{\mathrm{all}})\times 2,$$

where BV is the breeding value of the sire in a given trait, $x_{p}$ is the average performance of the sire's progeny group in that trait, and $X_{\mathrm{all}}$ is the average performance of the entire fattening-bull population tested in that trait.

In case of BLUP model, the animal model communicated the values of BV directly. The reliability value ( b ) of the estimated breeding values was calculated using the following formula (Tőzsér and Komlósi, 2004):

$$b=\sqrt{\frac{n\cdot R\cdot h^{2}}{\frac{1}{R}+(n-1)\cdot h^{2}}}$$

(Where: b = reliability value of BV; n = number of progeny of sire; $h^{2}$ = heritability of trait; R = degree of kinship.)

In the two different models, two different rankings were established based on the estimated BV of the sires based on the estimated traits. The effect of the model on the rank of sires was determined by Spearman's rank correlation (Spearman, 1904) calculation, similarly to in the study of Núnez-Dominguez et al. (1995).

Breeding values were determined for all sires, but in our paper, these are only shown (in a tabular form) for the 10 sires with the most offspring.

### 2.7 Estimation of genetic trends

The genetic trend of the examined traits – as in Ostler et al. (2005) – was determined from the average BV of sires born in the same year. Similarly to our previous work (Bene et al., 2021), calculations were performed using weighted one-way linear regression analysis. The mean BV was the dependent variable, the sire's year of birth was the independent variable, and and the number of offspring per sire was used for weighting.

## 3 Results

### 3.1 The effect of different factors

The effects of the sire, fattening farm, birth year of the fattening bulls and age of the bull progeny at slaughter on the 10 examined traits were shown in Table 2. The effect of the sire was found to be statistically significant ( p<0.01 ) for all traits. The effect of the age of the bull progeny at slaughter was significant for FFW, WDG, SLW, CAR, BMP and EUR traits. The effect of the fattening farm was significant on the examined traits (except for MUS). The effect of the birth year of the bulls was only detectable in the case of CAR, DRP and MEP traits.

**Table 2:** The effect of the factors on the estimated traits.

Traits		MUS	FFW	WDG	SLW	CAR	DRP	BMP	EUR	FAT	MEP
Sire	p	< 0.01	< 0.01	< 0.01	< 0.01	< 0.01	< 0.01	< 0.01	< 0.01	< 0.01	< 0.01
	%	24.55	2.24	0.43	2.75	2.72	22.49	0.53	7.63	7.98	15.85
Fattening farm	p	NS	< 0.01	< 0.01	< 0.01	< 0.01	< 0.01	< 0.01	< 0.01	< 0.01	< 0.01
	%	10.38	2.71	1.15	3.49	2.89	45.18	0.96	20.08	65.60	46.93
Birth year of	p	NS	NS	NS	NS	< 0.05	< 0.05	NS	NS	NS	< 0.01
bull	%	13.17	1.29	0.22	1.39	1.50	12.42	0.24	6.45	4.61	15.97
Age of bull at	p	NS	< 0.01	< 0.01	< 0.01	< 0.01	NS	< 0.01	< 0.01	NS	NS
slaughter	%	39.89	92.93	98.03	91.45	91.97	13.22	98.09	61.58	16.95	13.48
Error	%	12.01	0.83	0.17	0.92	0.92	6.69	0.18	4.26	4.86	7.77
Kolmogorov–Smirnov test ${}^{a}$	p	0.00	0.00	0.00	0.00	0.01	0.00	0.00	0.00	0.00	0.00
Levene test ${}^{b}$	p	0.00	0.00	0.00	0.00	0.00	0.00	0.00	0.00	0.00	0.20

${}^{a}$ If p>0.05 , the normal distribution is confirmed. ${}^{b}$ If p>0.01 , the homogeneity is confirmed. NS is for not significant, MUS is for muscularity score, FFW is for final fattening weight, WDG is for weight gain per day of life, SLW is for slaughter weight, CAR is for carcass weight, DRP is for dressing percentage, BMP is for bone–meat production per day of life, EUR is SEUROP conformation score, FAT is for SEUROP fat coverage score, and MEP is for meat percentage.

### 3.2 The mean of the examined traits

The averaged results of the progeny fattening bulls per year of birth for each trait are shown in Table 3. The corrected overall mean values of the examined traits were as follows: MUS – 6.8 points, FFW – 651.6 kg, WGD – 1217 g d ${}^{-1}$ , SLW – 611.1 kg, CAR – 369.3 kg, DRP – 60.4 %, BMP – 689 g d ${}^{-1}$ , EUR – 3.6 points (U-), FAT – 2.5 points and MEP – 70.6 %.

**Table 3:** The effect of the birth year of bull progeny on the estimated traits.

Year ${}^{a}$	N	MUS	FFW	WDG	SLW	CAR	DRP	BMP	EUR	FAT	MEP
		(point)	(kg)	(g d ${}^{-1}$ )	(kg)	(kg)	(%)	(g d ${}^{-1}$ )	(point)	(point)	(%)
2001	143	6.5	625.0	1183	603.1	359.3	59.5	680	3.2	2.3	70.5
2002	105	6.1	616.6	1166	594.5	355.3	59.7	673	3.5	2.1	72.0
2003	90	7.2	717.6	1255	683.6	413.6	60.7	731	3.3	2.8	68.5
2005	40	6.3	661.7	1183	623.9	372.6	59.5	667	4.0	2.0	68.7
2006	97	6.1	673.3	1201	632.7	383.9	60.5	686	3.2	2.6	69.2
2007	58	5.8	641.3	1169	598.4	351.9	58.7	639	3.1	2.3	69.3
2008	25	7.2	759.2	1373	707.7	427.7	59.9	775	4.1	2.4	69.8
2009	19	7.8	630.0	1146	599.5	373.1	62.2	681	4.4	2.6	72.7
2010	37	7.0	648.6	1223	612.2	371.0	60.7	700	3.7	2.7	71.0
2011	62	6.8	634.5	1212	595.6	353.5	59.4	674	3.9	2.8	68.4
2012	110	7.3	648.9	1247	602.9	364.0	60.5	698	3.9	2.6	71.2
2013	51	7.0	638.0	1229	587.3	360.0	61.2	690	3.7	2.5	71.1
2014	37	7.0	636.0	1206	587.4	374.5	63.4	705	3.5	2.7	72.9
2015	55	6.4	665.1	1250	614.9	376.4	61.4	705	3.7	2.8	71.4
2016	63	7.0	649.1	1228	598.7	359.7	60.1	676	3.5	2.8	70.8
2017	73	7.2	579.0	1126	538.8	327.8	60.8	635	3.5	2.5	71.5
2018	60	7.2	651.4	1267	608.6	364.3	60.0	707	3.7	2.6	71.0
2019	37	7.0	652.9	1249	609.3	358.7	58.9	684	3.7	2.7	70.2
Mean ${}^{b}$	1162	6.8	651.6	1217	611.1	369.3	60.4	689	3.6	2.5	70.6

${}^{a}$ Birth year of bull. ${}^{b}$ Corrected overall mean value. MUS is for muscularity score, FFW is for final fattening weight, WDG is for weight gain per day of life, SLW is for slaughter weight, CAR is for carcass weight, DRP is for dressing percentage, BMP is for bone–meat production per day of life, EUR is for SEUROP conformation score, FAT is for SEUROP fat coverage score, and MEP is for meat percentage.

### 3.3 Phenotypic trends and phenotypic correlations

According to the birth year of the bull progeny, significant differences were found in the examined traits (Table 3). The two extreme values of FFW were 579.0 kg (in 2017) and 759.2 kg (in 2008). Accordingly, we found a similarly large difference in the CAR (327.8 and 427.7 kg, respectively). In terms of WGD, 2009 was the worst (1146 g d ${}^{-1}$ ), and 2008 was the best (1373 g d ${}^{-1}$ ). The DRP exceeded 62 % in 2 years, 2009 and 2014.

**Table 4:** Phenotypic trend of the estimated traits based on the birth weight of bull progeny.

Traits	Slope ( bX )			Intercept ( a )			Fitting	
	b	SE	p	a	SE	p	$R^{2}$	p
MUS	+0.04	0.02	< 0.05	- 78.49	38.02	< 0.05	0.24	< 0.05
FFW	-1.18	1.54	NS	3017.80	3087.12	NS	0.04	NS
WDG	+2.06	1.70	NS	-2929.32	3417.31	NS	0.08	NS
SLW	-2.38	1.37	< 0.10	5399.18	2751.76	< 0.10	0.16	< 0.10
CAR	-1.20	0.88	NS	2786.20	1761.04	NS	0.11	NS
DRP	+0.05	0.03	< 0.10	-50.17	57.70	NS	0.19	< 0.10
BMP	-0.29	1.13	NS	1263.08	2273.01	NS	0.00	NS
EUR	+0.03	0.01	< 0.05	-49.81	20.15	< 0.05	0.30	< 0.05
FAT	+0.03	0.01	< 0.05	-48.20	18.61	< 0.05	0.32	< 0.05
MEP	+0.07	0.05	NS	-76.24	106.27	NS	0.11	NS

SE is the standard error, X is the birth year of the bull, MUS is the muscularity score (point), FFW is the final fattening weight (kg), WDG is the weight gain per day of life (g d ${}^{-1}$ ), SLW is the slaughter weight (kg), CAR is the carcass weight (kg), DRP is the dressing percentage (%), BMP is the bone–meat production per day of life (g d ${}^{-1}$ ), EUR is the SEUROP conformation score (point), FAT is the SEUROP fat coverage score (point), and MEP is the meat percentage (%).

**Table 5:** Phenotypic correlations between the estimated traits.

$r_{p}$	SLA	MUS	FFW	WDG	SLW	CAR	DRP	BMP	EUR	FAT	MEP
BYB	+0.03	${}^{a}+$ 0.19	${}^{a}+$ 0.29	${}^{a}+$ 0.20	${}^{a}+$ 0.15	${}^{a}+$ 0.26	${}^{a}+$ 0.34	${}^{a}+$ 0.19	${}^{a}+$ 0.24	${}^{a}-$ 0.14	${}^{a}+$ 0.34
SLA		${}^{a}+$ 0.15	${}^{a}+$ 0.43	${}^{a}-$ 0.68	${}^{a}+$ 0.43	${}^{a}+$ 0.44	${}^{a}+$ 0.11	${}^{a}-$ 0.66	${}^{a}+$ 0.15	${}^{a}-$ 0.16	${}^{a}+$ 0.14
MUS			${}^{a}+$ 0.43	${}^{a}+$ 0.19	${}^{a}+$ 0.43	${}^{a}+$ 0.48	${}^{a}+$ 0.19	${}^{a}+$ 0.24	${}^{a}+$ 0.44	+0.05	${}^{a}+$ 0.18
FFW				${}^{a}+$ 0.35	${}^{a}+$ 0.98	${}^{a}+$ 0.93	+0.04	${}^{a}+$ 0.32	${}^{a}+$ 0.37	+0.06	+0.03
WDG					${}^{a}+$ 0.34	${}^{a}+$ 0.29	${}^{a}-$ 0.10	${}^{a}+$ 0.96	${}^{a}+$ 0.13	${}^{a}+$ 0.22	${}^{a}-$ 0.12
SLW						${}^{a}+$ 0.93	-0.04	${}^{a}+$ 0.32	${}^{a}+$ 0.35	${}^{a}+$ 0.08	-0.02
CAR							${}^{a}+$ 0.34	${}^{a}+$ 0.36	${}^{a}+$ 0.44	+0.03	${}^{a}+$ 0.24
DRP								${}^{a}+$ 0.15	${}^{a}+$ 0.26	${}^{a}-$ 0.13	${}^{a}+$ 0.71
BMP									${}^{a}+$ 0.19	${}^{a}+$ 0.20	+0.05
EUR										${}^{b}-$ 0.08	${}^{a}+$ 0.32
FAT											${}^{a}-$ 0.45

In the above, $r_{p}$ is the phenotypic correlation. ${}^{a}$ p<0.01 . ${}^{b}$ p<0.05 . BYB is the birth year of the bull, SLA is the slaughter age of bull, MUS is the muscularity score, FFW is the final fattening weight, WDG is the weight gain per day of life, SLW is the slaughter weight, CAR is the carcass weight, DRP is the dressing percentage, BMP is the bone–meat production per day of life, EUR is the SEUROP conformation score, FAT is the SEUROP fat coverage score, and MEP is the meat percentage.

Statistically reliable ( p<0.05 ) phenotypic trends were found for only three traits, MUS, EUR and FAT (Table 4). In the case of these traits, the slope ( b ) had a positive sign so that we could observe a small increasing trend in these traits every year ( + 0.03 point yr ${}^{-1}$ ). In the case of the other traits, the slope value was not significant.

Based on the results of the phenotypic correlation calculation (Table 5), it appears that there was no close relationship between the studied traits, with a few exceptions. Such an exception was, for example, the negative, moderately strong correlation between SLA, WGD and BMP ( r = -0.68 , p<0.01 and r=-0.66 , p<0.01 ). The closest relationship was found between the FFF, SLW and CAR ( r = 0.93–0.98, p<0.01 ).

### 3.4 Population genetic parameters

The calculated population genetic parameters of the studied traits are summarized in Table 6. When estimated with GLM method, the MUS, the EUR, the FAT and the MEP showed moderate heritability ( $h^{2}$ = 0.23–0.32). The heritability value of the other traits was even higher ( $h^{2}$ = 0.42–0.53). Based on the SE (standard error) values, the statistical reliability of the $h^{2}$ values was satisfactory.

When estimated with the BLUP animal model, the heritability values were higher than what was experienced in the case of the GLM method.

**Table 6:** Population genetic parameters of the examined traits.

Traits	BLUP animal model				GLM model			
	$\sigma_{d}^{2}$	$\sigma_{e}^{2}$	$\sigma_{p}^{2}$	$h^{2}$ ± SE	$\sigma_{d}^{2}$	$\sigma_{e}^{2}$	$\sigma_{p}^{2}$	$h^{2}$ ± SE
MUS	0.5	0.7	1.2	0.42 ± 0.10	0.5	1.1	1.6	0.32 ± 0.09
FFW	2336.1	1468.4	3804.5	0.61 ± 0.11	2423.7	3168.8	5592.5	0.43 ± 0.10
WDG	8159.5	4975.0	13 134.5	0.63 ± 0.12	7796.3	10 808.3	18 604.6	0.42 ± 0.11
SLW	2263.6	1073.4	3337.0	0.68 ± 0.11	2441.0	2714.4	5155.4	0.47 ± 0.11
CAR	679.4	568.9	1248.3	0.54 ± 0.27	945.5	1066.3	2011.8	0.47 ± 0.14
DRP	3.5	1.5	5.0	0.70 ± 0.11	4.1	3.8	7.9	0.52 ± 0.10
BMP	3359.0	1157.3	4516.3	0.74 ± 0.12	3233.0	3656.7	6889.7	0.47 ± 0.10
EUR	0.1	0.2	0.3	0.34 ± 0.10	0.1	0.3	0.4	0.26 ± 0.09
FAT	0.1	0.1	0.2	0.32 ± 0.10	0.1	0.2	0.2	0.23 ± 0.09
MEP	1.6	3.5	5.1	0.31 ± 0.08	2.2	4.6	6.8	0.32 ± 0.09

$\sigma_{d}^{2}$ is the additive direct genetic variance, $\sigma_{e}^{2}$ is the residual variance, $\sigma_{p}^{2}$ is the phenotypic variance, $h^{2}$ is the heritability, MUS is the muscularity score, FFW is the final fattening weight, WDG is the weight gain per day of life, SLW is the slaughter weight, CAR is the carcass weight, DRP is the dressing percentage, BMP is the bone–meat production per day of life, EUR is the SEUROP conformation score, FAT is the SEUROP fat coverage score, and MEP is the meat percentage.

### 3.5 Breeding values and genetic trends

Tables 7 and 8 show the BV of sires as estimated by the BLUP animal model and the GLM method in terms of all the tested traits. For all traits, there was a significant difference in the estimated BV of the breeding bulls (sires).

**Table 7:** Breeding value in terms of the estimated traits for the sires with the most offspring.

Identity number	N	MUS		FFW		WDG		SLW		CAR	
of sire		(point)		(kg)		(g d ${}^{-1}$ )		(kg)		(kg)	
Mean ${}^{b}$	1162	6.8		651.6		1217		611.1		369.3	
GLM model		BV	SE	BV	SE	BV	SE	BV	SE	BV	SE
13 399	27	+0.6	0.2	+33.8	13.8	+57.6	26.9	+23.0	11.9	-5.8	7.0
15 669	17	+0.7	0.2	-46.0	18.1	-100.4	32.1	-57.2	17.1	-53.9	9.8
15 670	17	-0.6	0.2	-16.8	18.0	-57.5	30.9	-26.1	16.8	-43.7	9.9
16 931	17	-2.0	0.3	-29.3	19.0	+44.6	31.1	-35.6	16.8	-37.0	10.1
18 428	18	-1.9	0.2	-46.6	15.6	-37.6	30.9	-53.3	15.2	-39.9	8.6
19 227	31	-0.1	0.1	+22.2	11.0	+17.8	24.0	+13.8	10.0	-22.4	5.9
19 300	18	+0.4	0.2	+2.9	17.0	-7.2	30.6	-0.5	15.3	+1.2	8.4
21 521	22	+0.7	0.2	+6.0	14.1	-31.3	27.9	9.3	14.0	+6.2	7.0
22 658	20	-0.8	0.2	+32.0	14.8	+21.3	28.4	+24.6	14.3	+5.6	7.3
22 660	20	+0.0	0.2	+111.3	14.6	+159.5	28.6	+99.1	14.9	+53.7	7.3
BLUP model		BV	b	BV	b	BV	b	BV	b	BV	b
13 399	27	+0.6	0.66	+22.2	0.68	+49.6	0.68	+19.3	0.68	n.e.	n.e.
15669	17	+0.6	0.64	-44.2	0.66	-82.2	0.67	-49.1	0.67	n.e.	n.e.
15 670	17	-0.2	0.64	-14.8	0.66	-34.5	0.67	-17.4	0.67	n.e.	n.e.
16 931	17	-0.3	0.64	+36.4	0.66	+44.7	0.67	+31.8	0.67	n.e.	n.e.
18 428	18	-1.5	0.64	-60.3	0.67	-76.3	0.67	-66.6	0.67	n.e.	n.e.
19 227	31	-0.3	0.67	-5.1	0.68	-14.5	0.68	-11.5	0.69	n.e.	n.e.
19 300	18	+0.3	0.64	-23.2	0.67	-38.2	0.67	-23.7	0.67	n.e.	n.e.
21 521	22	+0.0	0.65	+39.0	0.67	+20.8	0.67	+37.5	0.68	n.e.	n.e.
22 658	20	-0.6	0.65	+3.1	0.67	-1.7	0.67	+0.7	0.68	n.e.	n.e.
22 660	20	+0.0	0.65	+60.0	0.67	+96.0	0.67	+54.9	0.68	n.e.	n.e.
$r_{\mathrm{rank}}$		${}^{a}$ 0.83		${}^{a}$ 0.79		${}^{a}$ 0.83		${}^{a}$ 0.82		n.e.	

${}^{a}$ <0.01 . ${}^{b}$ Corrected overall mean value. BV is the breeding value, SE is the standard error, b is the reliability value, N is the number of progeny, MUS is the muscularity score, FFW is the final fattening weight, WDG is the weight gain per day of life, SLW is the slaughter weight, CAR is the carcass weight, and n.e. means “cannot be estimated”.

**Table 8:** Breeding value in terms of the estimated traits for the sires with the most offspring.

Identity number	N	DRP		BMP		EUR		FAT		MEP	
of sire		(%)		(g d ${}^{-1}$ )		(point)		(point)		(%)	
Mean ${}^{b}$	1162	60.4		689		3.6		2.5		70.6	
GLM model		BV	SE	BV	SE	BV	SE	BV	SE	BV	SE
13 399	27	-3.1	0.4	-11.0	15.0	-0.2	0.1	+0.1	0.1	-4.1	0.3
15 669	17	-3.1	0.6	-108.5	18.0	+0.7	0.2	-0.3	0.1	-2.6	0.5
15 670	17	-4.5	0.6	-94.7	18.1	-0.7	0.1	-0.4	0.1	-4.1	0.6
16 931	17	-2.9	0.6	-28.5	17.9	-0.3	0.1	-0.5	0.1	+0.0	0.5
18 428	18	-1.5	0.6	-49.2	17.3	-0.6	0.1	-0.2	0.1	-1.2	0.4
19 227	31	-5.2	0.4	-48.8	12.1	-0.7	0.1	+0.1	0.1	-5.3	0.3
19 300	18	+0.1	0.6	-1.6	16.9	-0.1	0.1	+0.4	0.1	-0.9	0.5
21 521	22	+0.0	0.5	-8.0	16.0	+0.4	0.1	-0.6	0.1	+1.5	0.4
22 658	20	-1.7	0.6	-9.9	16.8	-0.5	0.1	-0.2	0.1	-1.2	0.5
22 660	20	-1.3	0.6	+73.0	16.6	-0.2	0.1	+0.6	0.1	-2.8	0.5
BLUP model		BV	b	BV	b	BV	b	BV	b	BV	b
13 399	27	-0.1	0.68	+25.4	0.69	-0.1	0.65	-0.2	0.65	-0.4	0.64
15 669	17	-0.5	0.67	-65.0	0.67	+0.4	0.62	-0.3	0.62	-0.1	0.62
15 670	17	-1.5	0.67	-40.2	0.67	-0.4	0.62	-0.4	0.62	-0.9	0.62
16 931	17	+1.2	0.67	+33.0	0.67	+0.0	0.62	-0.1	0.62	+0.4	0.62
18 428	18	-1.3	0.67	-69.7	0.68	-0.5	0.63	-0.3	0.62	-0.3	0.62
19 227	31	-5.2	0.69	-70.4	0.69	-0.5	0.66	+0.0	0.65	-4.5	0.65
19 300	18	-0.3	0.67	-25.1	0.68	+0.0	0.63	+0.1	0.62	-0.2	0.62
21 521	22	-2.1	0.68	-7.6	0.68	+0.0	0.64	+0.0	0.64	-0.8	0.63
22 658	20	-2.0	0.68	-23.8	0.68	-0.3	0.63	-0.1	0.63	-0.8	0.63
22 660	20	-1.7	0.68	+33.9	0.68	-0.1	0.63	+0.3	0.63	-1.8	0.63
$r_{\mathrm{rank}}$		${}^{a}$ 0.73		${}^{a}$ 0.86		${}^{a}$ 0.80		${}^{a}$ 0.63		${}^{a}$ 0.61	

${}^{a}$ <0.01 . ${}^{b}$ Corrected overall mean value. BV is the breeding value, SE is the standard error, b is the reliability value, N is the number of progeny, DRP is the dressing percentage, BMP is the bone-meat production per day of life, EUR is the SEUROP conformation score, FAT is the SEUROP fat coverage score, and MEP is the meat percentage.

When estimated with the GLM method, there was a difference of 260 g d ${}^{-1}$ between the best (registration no. 22660 sire, BV ${}_{\mathrm{WDG}}$ = +159.5 g d ${}^{-1}$ ) and the worst (reg. no. 15669 sire, BV ${}_{\mathrm{WDG}}$ = -100.4 g d ${}^{-1}$ ) sire in terms of WGD. The BV of the sire reg. no. 13399 was found to be the highest in the case of the MUS (BV ${}_{\mathrm{MUS}}$ = +0.8 points), which was an order of magnitude 2.6 points higher than that estimated in the case of the sire reg. no. 18428 (BV ${}_{\mathrm{MUS}}$ = -1.9 points).

The genetic trends obtained by averaging the estimated BV of the sires born in the same year in terms of 10 traits were summarized in Table 9. According to the results, for one trait, the change in the mean value over time ( b ) was negative for the FAT, and for the other traits, the slope ( b ) determined during the regression analysis was positive. It should be added to the positive results that the improvement of the average BV per trait from year to year was rather slow (e.g., in the case of WDG, the BV increased, on average, by only 0.70–0.72 g d ${}^{-1}$ yr ${}^{-1}$ ). In the case of the GLM method, for six traits, the reliability of genetic trends was statistically verifiable.

**Table 9:** Genetic trend of the estimated traits based on BV of sires.

Traits	Slope ( bX )			Intercept ( a )			Fitting	
	b	SE	p	a	SE	p	$R^{2}$	p
GLM method								
BV ${}_{\mathrm{MUS}}$	+0.06	0.02	< 0.05	-114.45	49.16	< 0.05	0.24	< 0.05
BV ${}_{\mathrm{FFW}}$	+1.78	2.75	NS	-3577.37	5511.59	NS	0.02	NS
BV ${}_{\mathrm{WDG}}$	+0.70	4.71	NS	-1407.77	9460.59	NS	0.00	NS
BV ${}_{\mathrm{SLW}}$	+1.63	2.48	NS	-3270.88	4980.13	NS	0.02	NS
BV ${}_{\mathrm{CAR}}$	+3.01	1.24	< 0.05	-6041.92	2490.58	< 0.05	0.26	< 0.05
BV ${}_{\mathrm{DRP}}$	+0.34	0.08	< 0.01	-672.72	167.05	< 0.01	0.49	< 0.01
BV ${}_{\mathrm{BMP}}$	+4.33	2.17	< 0.10	-8690.56	4357.35	< 0.10	0.19	< 0.10
BV ${}_{\mathrm{EUR}}$	+0.03	0.01	< 0.05	-63.38	28.67	< 0.05	0.22	< 0.05
BV ${}_{\mathrm{FAT}}$	-0.06	0.02	< 0.01	117.12	34.75	< 0.01	0.40	< 0.01
BV ${}_{\mathrm{MEP}}$	+0.27	0.08	< 0.01	-532.25	164.43	< 0.01	0.38	< 0.01
BLUP animal model								
BV ${}_{\mathrm{MUS}}$	+0.01	0.01	NS	-14.90	12.19	NS	0.08	NS
BV ${}_{\mathrm{FFW}}$	+0.41	0.76	NS	-839.41	1524.13	NS	0.02	NS
BV ${}_{\mathrm{WDG}}$	+0.72	1.34	NS	-1446.84	2679.81	NS	0.02	NS
BV ${}_{\mathrm{SLW}}$	+0.35	0.76	NS	-703.35	1518.87	NS	0.01	NS
BV ${}_{\mathrm{CAR}}$	n.e.	n.e.	n.e.	n.e.	n.e.	n.e.	n.e.	n.e.
BV ${}_{\mathrm{DRP}}$	+0.04	0.02	< 0.10	-81.16	46.40	< 0.10	0.15	< 0.10
BV ${}_{\mathrm{BMP}}$	+0.80	0.76	NS	-1597.29	1525.74	NS	0.06	NS
BV ${}_{\mathrm{EUR}}$	+0.00	0.00	NS	-4.85	4.71	NS	0.06	NS
BV ${}_{\mathrm{FAT}}$	-0.00	0.00	NS	5.39	4.45	NS	0.08	NS
BV ${}_{\mathrm{MEP}}$	+0.01	0.01	NS	-26.64	22.49	NS	0.08	NS

X is the birth year of the sire, BV ${}_{\mathrm{MUS}}$ is the BV of the muscularity score (point), BV ${}_{\mathrm{FFW}}$ is the BV of the final fattening weight (kg), BV ${}_{\mathrm{WDG}}$ is the BV of the weight gain per day of life (g d ${}^{-1}$ ), BV ${}_{\mathrm{SLW}}$ is the BV of slaughter weight (kg), BV ${}_{\mathrm{CAR}}$ is the BV of the carcass weight (kg), BV ${}_{\mathrm{DRP}}$ is the BV of the dressing percentage (%), BV ${}_{\mathrm{BMP}}$ is the BV of the bone–meat production per day of life (g d ${}^{-1}$ ), BV ${}_{\mathrm{EUR}}$ is the BV of the SEUROP conformation score (point), BV ${}_{\mathrm{FAT}}$ is the BV of the SEUROP fat coverage score (point), BV ${}_{\mathrm{MEP}}$ is the BV of the meat percentage (%), and n.e. means “cannot be estimated”.

## 4 Discussion

By comparing our results with the literature data, it can be established that the relevant sources report a lower FFW (419 kg; Özlütürk et al., 2004), a higher WDG (1338 g d ${}^{-1}$ ; Bureš and Bartoň, 2018) and a lower DRP (55.9 %; Coyne et al., 2019) than we experienced. The results of Gregory et al. (1994) were similar to ours, while Laborde et al. (2001) published significantly different SLW (659 kg) and CAR (405 kg) values. The MUS in our study was higher than that reported by Török et al. (2021).

Based on the results of our study, it can be stated again that the fattening and slaughter performances of Hungarian Simmental fattening bulls change significantly with age. A significant extension of the age at slaughter is accompanied by a decrease in weight gain and an increase in fat cover. This increases the live weight at slaughter, but if the muscularity is weaker, bone–meat production can drop drastically. Similarly to the data in the existing literature (Gregory et al., 1995; Lee et al., 1997; Honig et al., 2020), it can be concluded that the studied factors significantly influenced the fattening and slaughter results of fattening bulls.

The results of the phenotypic trend calculation fell short of our preliminary expectations. Earlier (Bene et al., 2016), we observed a small increase in the phenotypic trend of the fattening and slaughter characteristics of Hungarian Simmental bulls; the results of the present study showed a more stagnant state. The present results were different from those of Füller et al. (2009), who also observed a slight upward trend.

After the end of the tests, the meat of the fattened and slaughtered progeny was sold in the same way as their counterparts from normal fattening. As a result, the market and market trends and demands could inevitably have an influence on the evolution of certain traits, especially in the perspective of the examined 20-year time interval. Based on our results, it seems that, due to the changing market conditions, the FFW of the male progeny has decreased somewhat in the recent period. Lower finishing weight resulted in lower SLW and lower CAR, the reduction of which was also reflected in the phenotypic trends.

The worst inherited trait ( $h^{2}=0.23$ ) was the FAT, which was largely consistent with well-known professional axioms about the relationship between fat content and feeding (as an environmental factor). The heritability values obtained for the other traits were similar to those found in the literature (Crews et al., 2003; Hickey et al., 2007; Rumph et al., 2007; Su et al., 2017; Coyne et al., 2019).

Consistently with the results of our previous study (Bene et al., 2016), we can conclude that the sire of fattening bulls (breeding bulls under classification) can have a significant influence on the evaluated fattening and slaughter characteristics. We found greater differences in the BV of the sires in the study of some traits (for example, CAR or DWG) and smaller difference in the study of other traits (for example, DRP or FAT). Based on the results of our work, it can be repeatedly stated that, by choosing a suitable sire, it is possible to significantly improve the fattening and cutting performances even within one generation.

In the case of most of the examined traits, the genetic trends clearly showed an improvement in terms of the quality and BV of the sires used for breeding. The highest determination coefficients were shown for the DRP ( $R^{2}=0.49$ and 0.15; p<0.01 ) and the FAT ( $R^{2}=0.40$ and 0.08; p<0.01 ) traits. Similarly to our results, several sources (Potočnik et al., 2007; Emmerling et al., 2019) reported an increase in genetic trends. However, Kapš et al. (2000) and Röhrmoser and Pichler (2002) found the genetic trend of DWG to be stagnant.

For MUS, WDG, DRP, EUR and MEP, the phenotypic and genetic trends were of similar magnitude and direction (very small improvement). The genetic trend of FAT and BMP showed a minimal increase of a few grams per year, which was not reflected in the phenotypic trends. The evolution of these two traits can be considered to be stagnant from both a phenotypic and a genetic point of view.

The phenotypic and genetic trends of the FFW were in opposite directions. Presumably due to market changes, the offspring were fattened to a higher final weight in some years and to a lower final weight during the 20-year study period, which resulted in an overall decreasing phenotypic trend ( -1.18 kg yr ${}^{-1}$ ). At the same time, in the last quarter of the examined time interval, the progeny of several breeding bulls (e.g., reg. nos. 21521 and 22660) were also included in the progeny performance tests, with exceptionally high breeding values for the FFW. This appeared as an increase in the evolution of the genetic trend. Due to the very close correlation values ( r = 0.93–0.98, p<0.01 ), the findings for FFW were also valid for SLW and CAR.

Although the genomic breeding-value estimation and genomic selection are becoming more and more widespread in the breeding of the Simmental breed, the traditional performance test will continue to be present. In the earlier stage of our investigation, there were no snp (single-nucleotide polymorphism) data available; thus, the examination cannot cover them. However, the continuation of the work will be the examination of the snp effects in order to make a genomic estimation even more accurate in the future.

## 5 Conclusion

Individuals of the Simmental breed group are generally good meat producers, but their milk production is much weaker than that of dairy breeds. As a result, the focus with the Hungarian Simmental breed was mainly on the improvement of milk production capacity and secondary characteristics; thus, in the case of meat production capacity, maintenance or even a slight improvement can be considered to be an acceptable result. The latter statement is fully supported by the results of our present study.

The meat-breeding value index represents 27 % of the dual-purpose production index mentioned in the introduction. The meat-breeding value index is currently made up of the breeding value of three parameters – these are the bone–meat production, the prime meat percentage and the SEUROP conformation score (Húth el al., 2013; Kovács-Mesterházy, 2021). Based on our results, it seems that the simultaneous selection in which, in addition to milk production, meat production is included in the dual-production index at a proportion of almost one-third causes neither a substantial improvement nor a deterioration in the meat production of the Hungarian Simmental breed.

## Data Availability

Data are available from the corresponding author upon request.
